# Characteristics and comparisons of acute stroke in “recovered" to “active COVID-19 and “pre-pandemic” in Qatar database

**DOI:** 10.1007/s11239-021-02581-6

**Published:** 2022-03-04

**Authors:** Naveed Akhtar, Fatma Abid, Rajvir Singh, Saadat Kamran, Yahia Imam, Salman Al Jerdi, Sarah Salameh, Rand Al Attar, Muhammad Yasir, Hammad Shabir, Deborah Morgan, Sujatha Joseph, Muna AlMaslamani, Ashfaq Shuaib

**Affiliations:** 1 The Neuroscience Institute, Hamad Medical Corporation, Doha, Qatar; 2 Infectious Diseases Section, Medicine Department, Hamad Medical Corporation, Doha, Qatar; 3 Cardiology Research, Hamad Medical Corporation, Doha, Qatar; 4 Weill Cornell Medical College, Doha, Qatar; 5 Emergency Department, Hamad Medical Corporation, Doha, Qatar; 6 Medicine Department, Hamad Medical Corporation, Doha, Qatar; 7 Department of Medicine, University of Alberta, Edmonton, AB Canada

**Keywords:** Ischemic stroke, COVID-19, Outcome, Stroke severity, Post-Covid

## Abstract

Understanding the relationship of COVID-19 to stroke is important. We compare characteristics of pre-pandemic stroke (PPS), cases in acute COVID infection (CS) and in patients who have recovered from COVID-19 infection (RCS). We interrogated the Qatar stroke database for all stroke admissions between Jan 2020 and Feb 2021 (PPS) to CS and RCS to determine how COVID-19 affected ischemic stroke sub-types, clinical course, and outcomes prior to, during and post-pandemic peak. There were 3264 cases admitted (pre-pandemic: 3111, stroke in COVID-19: 60 and recovered COVID-19 stroke: 93). Patients with CS were significantly younger, had more severe symptoms, fever on presentation, more ICU admissions and poor stroke recovery at discharge when compared to PPS and RCS. Large vessel disease and cardioembolic disease was significantly higher in CS compared to PPS or RCS. There was a significant decline in stroke mimics in CS. Stroke in RCS has characteristics similar to PPS with no evidence of lasting effects of the virus on the short-term. However, CS is a more serious disease and tends to be more severe and have a poor prognosis.

## Highlights

- COVID-19- infection can result in endothelial injury and a prothrombotic state leading to an increased risk of stroke and other vascular diseases.
- It is not known if patients who recover from COVID-19 infection are also at an increased risk of stroke and whether their prognosis will be similar to stroke in patients with acute infection.
- We report a large series (3264 patients) with characteristics of symptoms and prognosis prior to, during and post-pandemic in a prospective registry.
- Our study shows that while stroke in patients with COVID-19 infection tended to be severe, stroke in patients who recovered from COVID-19 had similar characteristics and prognosis to pre-pandemic subjects.

## Introduction

The number of COVID-19 worldwide exceeded 121,000,000 as of March 17, 2021 with most patients recovering from the infection [1]. COVID-19 may affect the cardiovascular system and increases the risk of venous thrombosis and pulmonary embolism [2], myocardial injury [3] and stroke [4, 5]. Acute stroke has been reported in 0.5–2.5% of active COVID-19 and tends to be more severe with a higher mortality [4, 6]. To our knowledge, there are no reports of strokes in patients who have recovered from COVID-19.

We have previously published on acute stroke in COVID-19 pandemic from Qatar [7, 8]. Our main objective here was to compare historical strokes in pre-COVID-19 (PPS), active COVID-19 (CS) and strokes in recovered COVID-19 patients (RCS).

## Methods

The Qatar Stroke Database prospectively collects information on most acute stroke (98%) admitted in Qatar to the Hamad General Hospital (HGH) since February 2013 as previously published [9, 10]. The Institutional Review Board, Hamad Medical Corporation at the Medical Research Centre (MRC-01-20-489) approved the study. Data will be made available on request.

All acute stroke (AS) patients admitted to HGH between January-2019 to February-2020 were evaluated for the study (PPS) and served as the reference comparator for the COVID-19 cases. The CS cases all had active viral disease at the time of the stroke and the RCS patients had all recovered from the viral illness at the time of the stroke. The clinical information including risk factors, investigations, clinical presentation, and course during hospitalization were recorded. The severity of symptoms at admission (NIHSS score), clinical diagnosis as defined by the TOAST classification [11] and Bamford classification [12], and the length of stay in hospital are also recorded. The modified Rankin Scale (mRS) pre-admission, at discharge, and at 90-day follow-up are also documented.

### Patient and public involvement

Patients or the public WERE NOT involved in the design, or conduct, or reporting, or dissemination plans of our research.

### Statistical analysis

Descriptive statistics in the form of mean and standard deviations for continuous variables and frequency with percentages for categorical variables were performed. One-way ANOVAs with post hoc (Bonferroni) analyses were performed to see significant mean level differences for all continuous variables according to Pre-COVID, Active COVID and Post -COVID stroke groups. Chi-Square tests with standardized residuals were calculated to see association with categorical variables and the groups. Multivariate logistic regression analysis was performed to see associated risk factors to 90 days poor outcome. Adjusted odds ratio (OR) with 95% CI and P values were presented. P value less than equal to 0.05 (two tailed) was considered statistically significant level. SPSS 22.0 statistical package was used for the analysis.

## Results

There were 3264 patients [age; 52.8 ± 13.9 male/female 2404 (73.7%)/4860 (26.3%)] admitted to HGH during the study period and available for analysis. Of the 3264 stroke patients, there were 3111 patients admitted without COVID 19 in the 14 months prior to the pandemic (PPS), 60 cases with active COVID-19 infection (CS) and 93 COVID-19-recovered cases (RCS) as shown in the Table 1.

**Table 1:** Demographic and Clinical Characteristics of Patients with recovered COVID-19, active COVID-19, and pre-pandemic stroke patients

Characteristics or investigations	Total stroke cases (n = 3264)	Pre-COVID stroke (n = 3111)	Active-COVID stroke (n = 60)	Post-COVID stroke (n = 93)	P value
Age, Mean, years	52.8 ± 13.9	52.7 ± 13.9	53.4 ± 11.6	53.9 ± 13.8	0.63
Sex					
Male	2404 (73.7)	2277 (73.2)	56 (93.3)	71 (76.3)	0.002
Female	860 (26.3)	834 (26.8)	4 (6.7)	22 (23.7)	
Risk factors					
Hypertension	2123 (65.0)	2036 (65.4)	28 (46.7)	59 (63.4)	0.01
Diabetes	1473 (45.1)	1399 (45.0)	27 (45.0)	47 (50.5)	0.57
Dyslipidemia	1605 (49.2)	1579 (50.8)	4 (6.7)	22 (23.7)	< 0.001
Atrial Fibrillation on Admission	143 (4.4)	134 (4.3)	2 (3.3)	7 (7.5)	0.30
Active Smoking	823 (25.2)	798 (25.7)	10 (16.7)	15 (16.1)	0.04
Prior Stroke	392 (12.0)	375 (12.1)	7 (11.7)	10 (10.8)	0.93
BMI on admission	28.2 ± 5.4	28.2 ± 5.4	26.1 ± 3.7	27.6 ± 5.4	0.005
Fever on Admission	37 (1.1)	18 (0.6)	14 (23.3)	5 (5.4)	< 0.001
NIHSS on admission (mean)	3.8 ± 6.2	3.7 ± 6.1	8.9 ± 8.8	3.9 ± 6.2	0.001
NIHSS severity					
Mild (NIHSS 0–4)	2525 (77.4)	2427 (78.0)	27 (45.0)	71 (76.3)	< 0.001
Moderate (NIHSS 5–10)	367 (11.2)	342 (11.0)	14 (23.3)	11 (11.8)	
Severe (NIHSS > 10)	372 (11.4)	342 (11.0)	19 (31.7)	11 (11.8)	
Stroke treatment and course					
IV Thrombolysis given	159 (4.9)	153 (4.9)	(6.7)	2 (2.2)	0.38
Thrombectomy done	74 (2.3)	70 (2.3)	1 (1.7)	3 (3.2)	0.78
ICU Admission	225 (6.9)	202 (6.5)	17 (28.3)	6 (6.5)	< 0.001
Intubated during admission	172 (5.3)	158 (5.1)	11 (18.3)	3 (3.2)	< 0.0001
Final diagnosis					
Ischemic stroke	1370 (42.0)	1287 (41.4)	42 (70.0)	41 (44.1)	< 0.001
Intracerebral hemorrhage	270 (8.3)	260 (8.4)	1 (1.7)	9 (9.7)	
Cerebral venous thrombosis	43 (1.3)	37 (1.2)	4 (6.7)	2 (2.2)	
Transient ischemic attack	347 (10.6)	335 (10.8)	6 (10.0)	6 (6.5)	
Stroke mimics	1234 (37.8)	1192 (38.3)	7 (11.7)	35 (37.6)	
TOAST classification					
Small vessel disease	623 (44.1)	605 (45.7)	6 (12.8)	12 (27.9)	< 0.001
Large vessel disease	231 (16.3)	210 (15.9)	13 (27.7)	8 (18.6)	
Cardioembolic	359 (25.4)	329 (24.8)	16 (34.0)	14 (32.6)	
Stroke of determined origin	86 (6.1)	75 (5.7)	6 (12.8)	5 (11.6)	
Stroke of undetermined origin	115 (8.1)	105 (7.9)	6 (12.8)	4 (9.3)	
Prognosis at 90-Days (n = 2639)					
Good (mRS 0–2)	1941 (73.6)	1840 (74.0)	28 (46.7)	73 (78.5)	< 0.001
Poor (mRS 3–6)	698 (26.4)	646 (26.0)	32 (53.3)	20 (21.5)	
Mortality at 90-Days (n = 2639)	100 (3.8)	92 (3.7)	6 (10.0)	2 (2.2)	0.03

There was no significant difference in the age of the three groups. The higher percentage of males reflects the demographics of Qatar with a predominantly male expatriate population as have been previously reported [9, 10]. The mean duration of time between recovery from COVID-19 infection and stroke was 126.9 ± 75.9 days (median 124 days). There was a significant decrease in stroke mimics in the CS cases as compared to PPS and RCS. Small vessel disease (SVD) is the most common type of stroke in the Qatari and expatriate population, likely due to the high prevalence of poorly controlled hypertension and diabetes as has been previously documented [10]. SVD was significantly lower in CS (12.8%) compared to 45.7% in PPS and 27.9% in RCS (p < 0.001). The CS group was associated with an increase in the percentage of large vessel and embolic stroke as shown in the Table 1.

The CS patients were more likely to have higher NIHSS on admission and significantly more patients had cortical strokes. The admission NIHSS was 8.9 ± 8.8 in CS, compared to 3.7 ± 6.1 in the PPS and 3.9 ± 6.2 in RCS patients (p < 0.001). Active COVID-19 patients were more likely to be febrile (23.3% vs PPS (0.6%) and RCS (5.4%). Patients with active COVID-19 and stroke were more likely to have admissions to the ICU (CS: 28%, PPS: 6.5% and RCS: 6.5%; p < 0.001), more frequently required intubation (CS: 31.3%, PPS: 5.1% and RCS: 3.2%) and had longer length of hospitalization [LOC] (CS: 27.1 ± 23.1, PPS: 4.1 ± 7.2 and PCS: 4.7 ± 6.6 days)**.**

Patients with stroke following recovery from COVID-19 had a clinical profile very similar to PPS patients. They had milder disease on admission, and similar mRS at discharge (mRS [0–2] CS: 35%, PPS: 71.2% and RCS: 73% p < 0.0001). They were, however, more likely to be febrile on admission compared to PPS. Recovering COVID stroke patients with fever had similar clinical course and prognosis to patients without fever (poor outcome [mRS 3–6] 20.0 vs 21.6%, p = 0.93) and all febrile recovering stroke patients had no evidence of active COVID-19 infection.

**Fig. 1:**
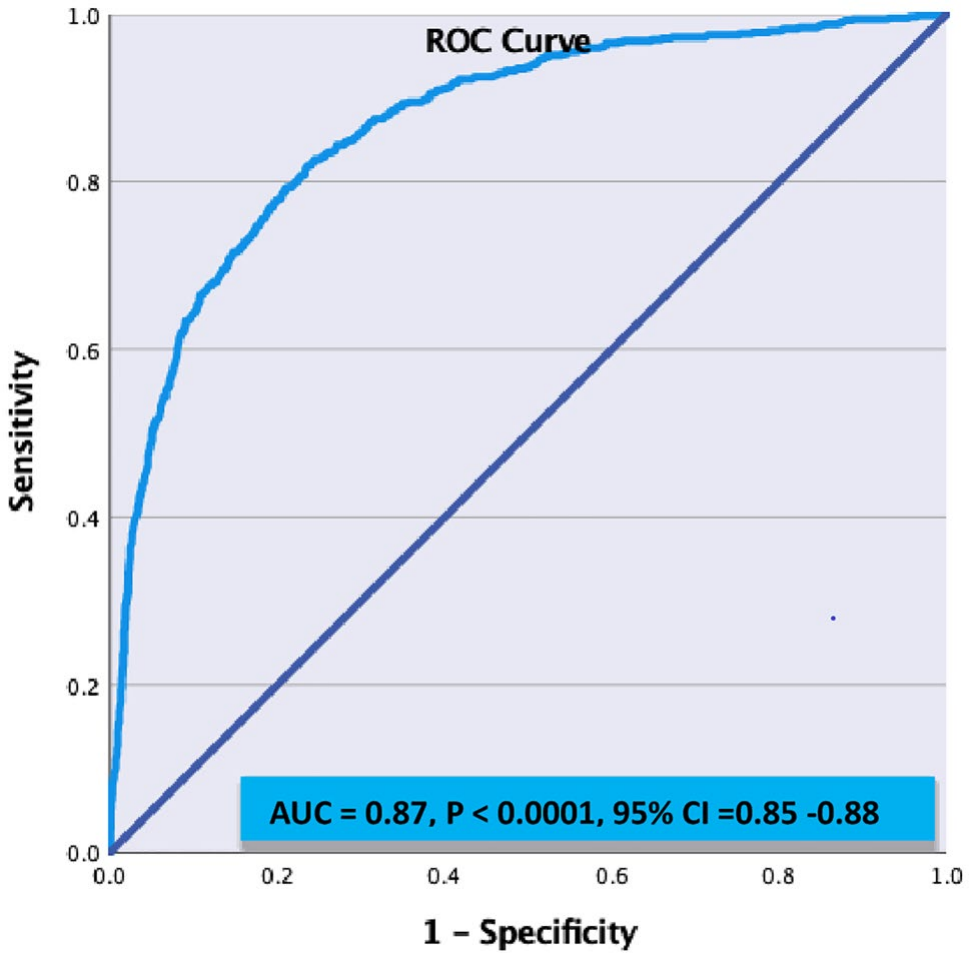
ROC CURVE- To predictive accuracy of 90-Day Prognosis from the model

Patients with active COVID-19 and stroke had slower recovery during hospitalization and at 90-days follow-up when compared to PPS or RCS patients (Fig. 1). At 90-day follow up, good recovery (mRS 0–2) was seen in 46.7% in CS patients compared to 74% in PPS and 78.5% in RCS subjects (p < 0.0001).

Adjusting age and sex in the multivariate logistic regression analysis, NIHSS score on admission (adjusted OR 1.23, 95% CI 1.20–1.26, p = 0.001) and febrile on admission (adjusted OR 3.65, 95% CI 1.36–9.83, p = 0.01), were found to be associated with poor outcome at 90 days (Table 2). There was no statistical association for BMI, prior hypertension, ICU admission and intubated during admission. The regression model was able to discriminate 87% accurately for the 90 days poor outcome.

**Table 2:** Multivariate analysis of the variables associated with 90-Day poor outcome in all three groups

Variable	Odds ratio	95% CI		P value
		Lower	Upper	
Age	1.08	1.07	1.09	< 0.001
Sex	0.66	0.52	0.84	< 0.001
BMI	0.66	0.99	1.03	0.42
Prior hypertension	1.27	0.97	1.68	0.08
Prior dyslipidemia	1.25	0.99	1.58	0.06
NIHSS score on admission	1.23	1.20	1.26	< 0.001
Febrile on admission	3.65	1.36	9.83	0.01
ICU admission	1.01	0.54	1.86	0.99
Intubated during admission	1.49	0.72	3.06	0.28

## Discussion

Similar to numerous previous reports, we also observed acute stroke in active COVID-19 patients to be severe, more likely to have cortical involvement and had relatively poor recovery when compared to PPS. Active COVID-19 related stroke patients were also more likely to be febrile, requiring intubation and ICU admissions, and longer hospital stay. The significantly lower rates of stroke mimics in the CS case likely relates to a fear of coming to hospital in the pandemic [8]. The most important new observation from our study relates to the stroke in patients with full recovery following COVID-19 infection. The overall pattern of stroke in this group appeared to have symptoms and clinical course similar to patients with stroke prior to the pandemic.

To our knowledge, this is the first study that compares stroke in active COVID-19 patients to non-COVID-19 patients and subjects who have recovered from the infection. It is important to note that we noticed no long-lasting effects of COVID-19 in any of our RCS patients. When comparing to the 3111 patients with no COVID-19, the RCS had identical presentation, risk factors, clinical course, and prognosis. It is also interesting that once the patients recovered, the stroke subtypes were very similar to what we had observed over the past 7 years [8].

Our study suggests that COVID-19 did not contribute to the etiology of stroke once the patient recovers. There are however several factors related to COVID-19 that may increase the risk of stroke in patients who have recovered and these needs attention [13, 14]. Potential mechanisms include continued endothelial injury [13], cardioembolism and potential paradoxical embolism via a PFO [15] or arterial dissection [16]. While the recovery is complete following COVID-19 in most patients, the “long-haulers” may have prolonged illness and therefore are at risk for complications [17]. COVID-19 infection results in injury to the arterial endothelium, resulting in a prothrombotic state [13]. The prothrombotic state may persist and increase the risk of stroke. Cardiac muscle injury and heart failure seen with COVID-19 [15] may potentially contribute to embolic stroke in some cases. Cardioembolism was the final diagnosis in 14% of our patients with stroke following recovery from COVID-19 which is lower than the 25% seen in pre-COVID-19 cases and therefore likely did not contribute to the post-COVID-19 cases. Similarly, there were no cases of arterial dissection in the post-COVID-19 group.

There are strengths to our study. The Qatar Stroke Database is very robust and has prospectively recorded stroke trends in the country for more than 7 years. While the prospective data collection had shown a steady increase in admission rates over several years, the dramatic decline during over three months as the number of COVID-19 cases is very striking [8]. This is similar to multiple observations from around the world. Our study shows that active COVID-19 positive stroke patients were more likely to be sicker, had more cortical involvement and had prolonged LOC and fewer frequency of good recovery at discharge. We also showed that patients who suffer a stroke following recovery from COVID-19 has similar characteristics to pre-COVID-19 cases.

The study has some limitations. A change over three months is brief and may not be sufficient to completely understand COVID-19-related changes. We did not document the relationship between the severity of COVID-19 and stroke. We also do not have enough long-term follow-up data at present on the patients seen during the pandemic to adequately document the changes in outcomes.

In summary, we present a comparison study on stroke subtypes prior to the pandemic to COVID-19 positive cases, and stroke in patients who recovered from the illness. Our data in 93 patients who had recovered from COVID-19 is reassuring in indicating no short-term effects of the illness.

## Data Availability

All relevant information is included in the manuscript. Any further data request can me made available on request.

## Abbreviations

PPS: Pre-pandemic stroke
CS: COVID infection stroke
RCS: Recovered from COVID-19 infection
